# ALOX5AP suppresses osteosarcoma progression via Wnt/β-catenin/EMT pathway and associates with clinical prognosis and immune infiltration

**DOI:** 10.1186/s13018-023-03919-x

**Published:** 2023-06-22

**Authors:** Guo-Dong Han, Jian Dai, Hong-Xia Hui, Jing Zhu

**Affiliations:** 1 Department of Orthopedics, The Affiliated Huaian No.1 People’s Hospital of Nanjing Medical University, Huaian, China; 2 Department of Medical Oncology, The Affiliated Huaian No.1 People’s Hospital of Nanjing Medical University, Huaian, China

**Keywords:** Osteosarcoma, Biomarker, Immune infiltration, Machine learning, Metastasis, ALOX5AP, WNT/β‐catenin signaling

## Abstract

Osteosarcoma (OS) is one of the most common malignant neoplasms in children and adolescents. Immune infiltration into the microenvironment of the tumor has a positive correlation with overall survival in patients with OS. The purpose of this study was to search for potential diagnostic markers that are involved in immune cell infiltration for OS. Patients with OS who acquired metastases within 5 years (*n* = 34) were compared to patients who did not develop metastases within 5 years (*n* = 19). Differentially expressed genes (DEGs) were tested for in both patient groups. To discover possible biomarkers, the LASSO regression model and the SVM–RFE analysis were both carried out. With the assistance of CIBERSORT, the compositional patterns of the 22 different types of immune cell fraction in OS were estimated. In this research, a total of 33 DEGs were obtained: 33 genes were significantly downregulated. Moreover, we identified six critical genes, including ALOX5AP, HLA-DOA, HLA-DMA, HLA-DRB4, HCLS1 and LOC647450. ROC assays confirmed their diagnostic value with AUC > 0.7. In addition, we found that the six critical genes were associated with immune infiltration. Then, we confirmed the expression of ALOX5AP was distinctly decreased in OS specimens and cell lines. High expression of ALOX5AP predicted an advanced clinical stage and overall survival of OS patients. Functionally, we found that overexpression of ALOX5AP distinctly suppressed the proliferation, migration, invasion and EMT via modulating Wnt/β‐catenin signaling. Overall, we found that ALOX5AP overexpression inhibits OS development via regulation of Wnt/β‐catenin signaling pathways, suggesting ALOX5AP as a novel molecular biomarker for enhanced therapy of OS.

## Introduction

Osteosarcoma (OS), one of the commonest primary malignant bone tumors that most commonly occur in children and teenagers, is prone to invasion and distant metastasis [1]. It displays osteoblastic differentiation and creates cancerous osteoid. It is characterized by the fact that it is a sarcoma [2]. Patients diagnosed with localized osteosarcoma who undergo the standard treatment protocol, including preoperative chemotherapy, surgery and postoperative adjuvant chemotherapy, have a five-year survival rate of 60–70% [3, 4]. On the other hand, the early clinical indications of OS are not easily recognizable or distinguishable. More than one fifth of OS patients already have lung metastases at the time of diagnosis, which renders the available treatment choices ineffective and drastically reduces the likelihood of survival for these patients to barely 15–20 percent [5, 6]. Patients who have advanced metastatic OS have a dismal outlook on their health. Patients with OS frequently build up a resistance to the traditional medications; hence, treatment regimens need to be modified, and new therapeutic targets need to be developed [7, 8]. The limited knowledge regarding the molecular mechanisms underlying OS hinders the progress of prognostic and therapeutic advancements. Therefore, it is imperative to identify additional diagnostic and prognostic biomarkers for OS.

Microarray technology, in conjunction with integrated bioinformatics research, has been utilized in recent years with the goal of finding novel genes associated with a variety of malignancies that have the potential to operate as diagnostic and prognostic biological markers [9, 10]. For instance, Li et al. reported that gliomas had significant levels of CNPY4 expression. Increased CNPY4 expression was found to be linked with tumor age, grade, the presence or absence of IDH, and 1p/19q codeletion, according to the results of an analysis using univariate logistic regression. Downregulation of CNPY4 expression was found to be an independent and satisfactory prognostic factor after multivariate analysis. There was a correlation between the degree of CNPY4 expression and the infiltration of dendritic cells in glioblastoma [11]. Gong and his group showed that an effective method for the prognostic classification of clear cell renal cell carcinoma is the TRPV family. TRPV3 is the most significant predictive predictor of clear cell renal cell carcinoma among these several other markers. In addition to this, they carried out a drug sensitivity study in order to determine which medicines had the most significant correlation with TRPV3. As a consequence of this, the TRPV family, and specifically TRPV3, can serve as a predictive biomarker in clear cell renal cell carcinoma to identify both the patient's prognosis and the degree to which immune cells have been infiltrated [12]. Recently, Li et al. performed the least absolute shrinkage and selection operator (LASSO) and support vector machine (SVM) machine learning methods, finding that Eleven key genes(PDK4, PYY, MMP11, MMP7, KLF4, TRIB3, FOXQ1, DPEPCFD, CFD, BEST4 and ASCL2) as novel diagnostic biomarkers for colorectal cancer [13]. In addition, the above genes were functionally confirmed in vitro and in vivo in several studies. However, the function of LASSO and SVM machine learning methods used in identifying diagnostic biomarkers for metastasis OS has not been reported.

The methods of machine learning were utilized in this study for the purpose of screening prospective diagnostic genes. In order to generate a diagnostic signature that is more reliable, we made use of the LASSO and SVM algorithms, which are two of the most widely used traditional machine learning approaches. Then, we demonstrated the diagnostic value of the novel biomarkers by the use of ROC assays. In addition, CIBERSORT analysis was employed to examine immune cell infiltration and establish connections between crucial genes and the invading immune cells within the tumor in order to identify new biomarkers for the diagnosis and treatment of OS. Finally, we focused on ALOX5AP and further explored its function in OS progression.

## Materials and methods

### Data collection and preprocessing

Downloaded from the GEO database, the gene expression data GSE21257 for 53 patients with OS were then corrected and annotated using R software. OS patients who developed metastases within 5 years accounted for 34 of the 53 patients, while patients who did not develop metastases within 5 years accounted for the remaining 19 individuals.

### Differentially expressed genes (DEGs) screening

Robust multi-array average (RMA) was applied to the raw expression data contained in the GSE21257 dataset in order to adjust and normalize the background. In high-throughput tests, the SVA package of the R soft programming language was removed because it was causing batch effects and other irrelevant factors. The “limma” package of the R software was able to detect the DEGs in both OS and normal tissues with a *P*-Value of less than 0.05 and an absolute logFC of greater than 1. Utilizing the edgeR package of the R software to standardize the GSE21257 datasets and screen for DEGs with *P*-Val values of less than 0.05 and absolute logFC values of more than 1.

### Functional enrichment analysis

The Gene Ontology (GO) analysis is a widespread and helpful method for annotating genes and gene products and for finding characteristic biological properties of high-throughput genome or transcriptome data. This method was developed by Gene Ontology Consortium (GO Consortium). The Kyoto Encyclopedia of Genes and Genomes (KEGG) is a database that is well known for its systematic analysis of gene activities in biological pathways. This database integrates genomic information with higher-order functional information. For the purpose of functional enrichment analysis, the "ClusterProfiler" package of the R software was utilized, and the GO biological processes and KEGG pathways that met the criteria for significance (*q*-value of at least 0.01) were utilized. DEGs were analyzed for disease ontology (DO) enrichment using the "clusterProfiler" and DOSE packages in the R programming language [14]. In order to provide fresh insights into interconnected illnesses, the DO classification employs a set of formal semantic criteria to define meaningful disease models that take into account multiple-inferred mechanistic disease categories [15].

### Machine learning algorithm model construction

In the field of biomedicine, promising biomarkers were screened with the help of machine learning algorithms. In order to construct diagnostic models and analyze gene signals, we make use of machine learning techniques. The objective of LASSO regression was to identify the variable result that yields the lowest prediction error and obtain the corresponding regression coefficient. When the regression coefficient is constrained, the result that is obtained is the best possible one. In order to get the most accurate value for lambda, the Glmnet package is utilized [16]. The SVM model, which has just recently become popular in the field of biomedicine, is another useful tool for identification, prediction, or classification. We can use them to pick the best variables for the advantages that can't be categorized by linear decision data, and RFE can categorize different characteristics. Both of these things are possible thanks to the RFE. In order to put the SVM–RFE algorithm into action, the e1071 package is required. By employing two distinct machine learning approaches, an exceptionally high level of accuracy was achieved in identifying the intersection gene as a diagnostic gene signal.

### Diagnostic value of feature biomarkers in OS

An ROC curve was created by using the mRNA expression data from 34 OS samples with metastasis and 19 OS samples without metastasis. This allowed us to determine whether or not the found biomarkers had any predictive value. The value of the AUC was used to measure the diagnostic efficiency in distinguishing between OS samples with metastases and OS samples without metastases, and this distinction was further confirmed using the GSE21257 dataset.

### Estimation of tumor immune microenvironment

In order to measure the relative abundance of 22 immune cells that had been infiltrated into the microenvironment of the tumor using a deconvolution method, the CIBERSORT algorithm was carried out. In order to assess the connection between the expression of essential genes and immune infiltration, the CIBERSORT method was implemented in the GSE21257 dataset.

### Patients and clinical specimens

Between January 2015 and December 2017, matched fresh osteosarcoma specimens and surrounding nontumorous tissues were obtained from thirty patients at The Affiliated Huaian No.1 People's Hospital of Nanjing Medical University. Every specimen was snap-frozen in liquid nitrogen as soon as it was collected, and it was kept at a temperature of -80 degrees Celsius until it was used. Each patient's follow-up data were accessible in their entirety and were complete. The day of the primary operation served as the starting point for the calculation of overall survival, which continued until the patient passed away or the study was discontinued. This project was approved by the Clinical Research Ethics Committee of our hospital and all patients provided written informed consent.

### Cell culture

Four human osteosarcoma cell lines (MG63, Saos-2, U-2OS and 143B) and human osteoblast hFOB1.19 cells were provided by the Cell Bank of the Chinese Academy of Sciences (Shanghai, China). At a temperature of 37 degrees Celsius and in a humidified environment containing 5% carbon dioxide, the cells were grown in Dulbecco's Modified Eagle's Medium that had been supplemented with 10% fetal bovine serum (FBS).

### Cell transfection

The overexpressing plasmids, including pcDNA3.1 and pcDNA3.1-ALOX5AP, were purchased from Genetong Biological corporation (Xiamen, Fujian, China). In line with the methods that were included in the Lipofectamine 2000 reagent kits, the cell transfection was carried out utilizing those kits. In order to evaluate the success of the transfection, quantitative real-time PCR was performed on the cells that had been previously transfected.

### Real-time reverse transcription-PCR

Trizol reagent (Invitrogen, Carlsbad, California, USA) was used to extract total RNA, and a RevertAid First Strand cDNA Synthesis Kit (Fermentas, Vilnius, Lithuania) was used in the cDNA synthesis process. Gene expression of ALOX5AP was monitored by real-time PCR using ‘Assays on Demand' (Applied Biosystems, Alameda, CA, USA). The values of expression were normalized by comparing them to the geometric mean of GAPDH. The primers that used to amplify the cDNA were as follows: F: 5′-TCAGCGTGGTCCAGAATGG-3′, R: 5′-GCAAGTGTTCCGGTCCTCT-3′ for ALOX5AP; and F: 5′-GGAGCGAGATCCCTCCAAAAT-3′, R: 5′-GGCTGTTGTCATACTTCTCATGG -3′ for GAPDH.

### Proliferation assay

In order to monitor cell growth, a Cell Counting Kit-8 (CCK-8, manufactured by Beyotime in Shanghai, China) was utilized. Briefly, following a transfection period of forty-eight hours, various types of cells were seeded into 96-well plates. Incubation of the cells in CCK-8 solution was placed for one hour on days 1, 2 and 3. After that, an optical density (OD) reading was taken at 450 nm using a microplate reader. The trials were carried out three times in all.

### Colony formation assays

In order to perform the colony formation experiment, each batch of cells was seeded onto six-well plates at a low density of 500 cells per well. The plates were subsequently incubated for a duration of ten days. Following that, the cells were stained using a crystal violet solution (Sigma) consisting of 0.5% crystal violet. After giving the cells a quick rinse to remove any debris, digital photographs were taken of them using a microscope manufactured by Olympus Inc., and the number of colonies that were visible was recorded.

### EdU incorporation analysis

This experiment was carried out with the use of a BeyoClickTM EdU Cell Proliferation Kit (Beyotime, Shanghai, China), which was used in accordance with the procedures provided by the provider. After placing 1 × 10^4^ cells in the 96-well plate, the cells were treated with an EdU kit at room temperature for a period of two hours. After that, cells were stained with DAPI for 5 min. In the end, cells were viewed through a fluorescence microscope manufactured by Olympus in Tokyo, Japan. The biological replicates were carried out three times.

### TUNEL staining

After being transfected, the cells were grown for the following 24 h. After being washed twice with PBS, the cells were fixed with 4% paraformaldehyde for 15 min and then permeabilized with 0.25% Triton-X 100 for 20 min. TUNEL tests were carried out in a manner that was consistent with the instructions provided by the manufacturer (Roche). First, the cells were treated with a Click-iT reaction cocktail after being incubated in a terminal deoxynucleotidyl transferase (TdT) reaction cocktail for 45 min at 37 degrees Celsius. Subsequently, the cells were washed, and either hematoxylin or methyl green was employed to achieve the desired nuclear staining.

### Cell migration and invasion assays

In order to determine the cell migration, a wound-healing test was carried out. To summarize, a sterile pipette tip with 200 μL was used to scrape transfected cells that had been grown to 90% confluence in 6-well plates. After cells had been cultured for 24 h and washed with serum-free medium to remove any debris that may have been present, images were acquired using an inverted microscope (TE2000, manufactured by Nikon in China). Calculations were done to determine the wound-healing rates, and the results were compared to the breadth of the wound at 0 h. In order to evaluate the cell invasion, a Transwell assay was carried out using Matrigel Invasion Chambers. These procedures were carried out in accordance with the instructions provided by the manufacturer (BD Biosciences). In a nutshell, the cells were spread out throughout the top surface of the Transwell insert. After a period of 24 h, the invasive cells were stained with 0.5% crystal violet and then fixed with 4% paraformaldehyde. The count of invasive cells was performed in five randomly selected microscope views, and photographs were captured to document the results.

### Animal treatment

BALB/c mice (female, 4–6 weeks of age, 18–20 g; Shanghai SLAC Laboratory Animal Co., Ltd.) were maintained in an environment free of pathogens and were allowed to consume food and acidified water at their own discretion. Each group consisted of six mice, resulting in a total of twelve mice in the study. Each group received a distinct treatment, and this process was repeated twelve times. The size of the sample was decided upon after taking into consideration statistical power and ethical constraints. Independent evaluations were performed in accordance with the rules provided by the AAA-LAC to determine the inclusion and exclusion criteria for the animals. Subcutaneous injections of a mixture containing 2.0 × 10^7^ MG63 and Saos-2 cells in 100 μL PBS were given to the animals in each group. All experiments on animals were carried out in accordance with the Guidelines for the Care and Use of Laboratory Animals, and the Institutional Animal Care and Use Committee at The Affiliated Huaian No.1 People's Hospital of Nanjing Medical University gave their approval for the experiments to be carried out.

### Western blot analysis

The cells were lysed in a radioimmunoprecipitation assay buffer provided by the Beyotime Institute of Biotechnology in Nantong, China, along with a protease inhibitor cocktail provided by Thermo Fisher Scientific, Inc. The total protein extracts were quantified using a Bicinchoninic Acid assay kit provided by Thermo Fisher Scientific, Inc., following the manufacturer's instructions. After being separated by 12% SDS-PAGE, a total of 40 g of protein was then transferred to a polyvinylidene fluoride membrane (EMD Millipore). Following blocking in 5% milk for one hour at room temperature, proteins were detected using specific primary antibodies against β-catenin, E-cadherin, N-cadherin, MMP9 and GAPDH. These antibodies were incubated at 4 degrees Celsius overnight. The membrane was treated with a goat anti-rabbit IgG-HRP secondary antibody at room temperature for one hour. Enhanced chemiluminescence was used to view the proteins, and ImageJ was used to perform densitometry calculations on the bands in order to determine their relative abundance. All antibodies were purchased from Abcam (Pudong, Shanghai, China).

### Statistical analysis

All statistical analyses were conducted using R (version 3.6.3). Continuous variables were expressed as mean ± standard deviation. Comparisons between two independent groups were analyzed by Student’s t test. ROC assays were carried out to explore the diagnostic value of the novel biomarkers. Both the Kaplan–Meier technique and the log-rank test were utilized in the plotting of survival curves. Cox proportional-hazards modeling was employed for both univariate and multivariate analysis to assess the influence of various factors on survival. *P* < 0.05 was considered statistically significant.

## Results

### Identification of DEGs between OS samples with metastases and OS samples with non-metastases

**Fig. 1:**
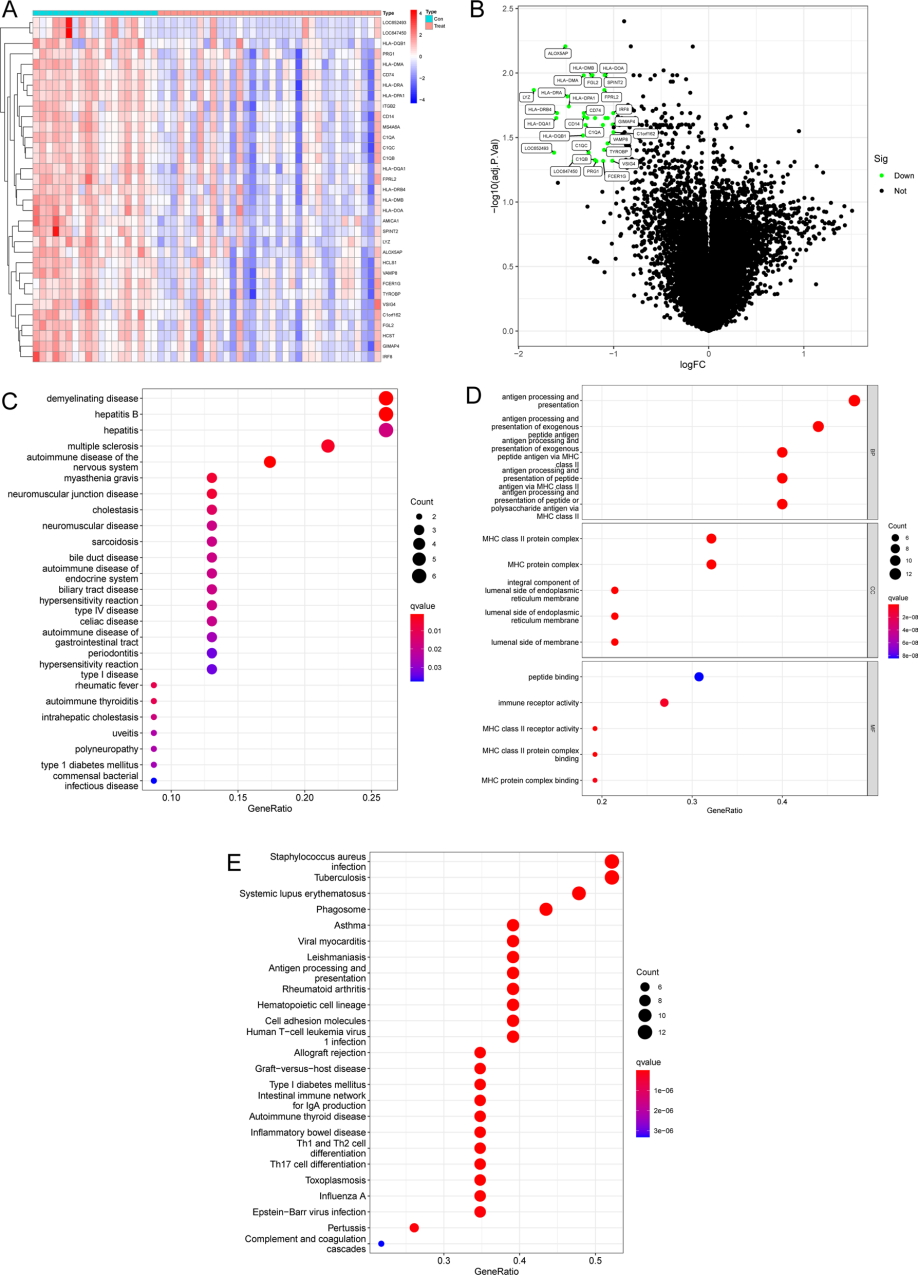
Differentially expressed genes between OS samples with metastasis and OS samples with non-metastasis. **A** On the heat map of genes with differential expression, highly expressed genes are shown in red, while genes with low expression are shown in blue. **B** The volcanic map of genes that have variable levels of expression. Fold changes greater than one are shown by either red (indicating upregulation) or green (downregulation). **C** Disease assays. **D** GO function assays. **E** KEGG pathway assays. BP, biological processes; CC, cellular component; MF, molecular function

Data from a total of 34 OS samples with metastases and 19 OS samples with non-metastases from GSE21257 datasets were analyzed. The DEGs were analyzed using the limma package. 33 DEGs were collected: All 33 genes were significantly downregulated (Fig. 1A and B)

### Functional correlation analysis

To learn more about how DEGs work, we performed pathway enrichment studies using DO. We observed that diseases enriched by DEGs were mainly related to demyelinating disease, hepatitis B, hepatitis, multiple sclerosis, autoimmune disease of the nervous system, myasthenia gravis, neuromuscular junction disease, cholestasis and neuromuscular disease (Fig. 1C). The results of GO analysis revealed that the 53 genes were mainly associated with antigen processing and presentation, MHC class II protein complex, MHC protein complex, peptide binding, immune receptor activity and MHC class II receptor activity (Fig. 1D). Moreover, in KEGG assays, we observed that the 53 genes were mainly enriched in Staphylococcus aureus infection, Tuberculosis, systemic lupus erythematosus, phagosome, inflammatory bowel disease and Th17 cell differentiation (Fig. 1E).

### Identification and validation of diagnostic feature biomarkers

**Fig. 2:**
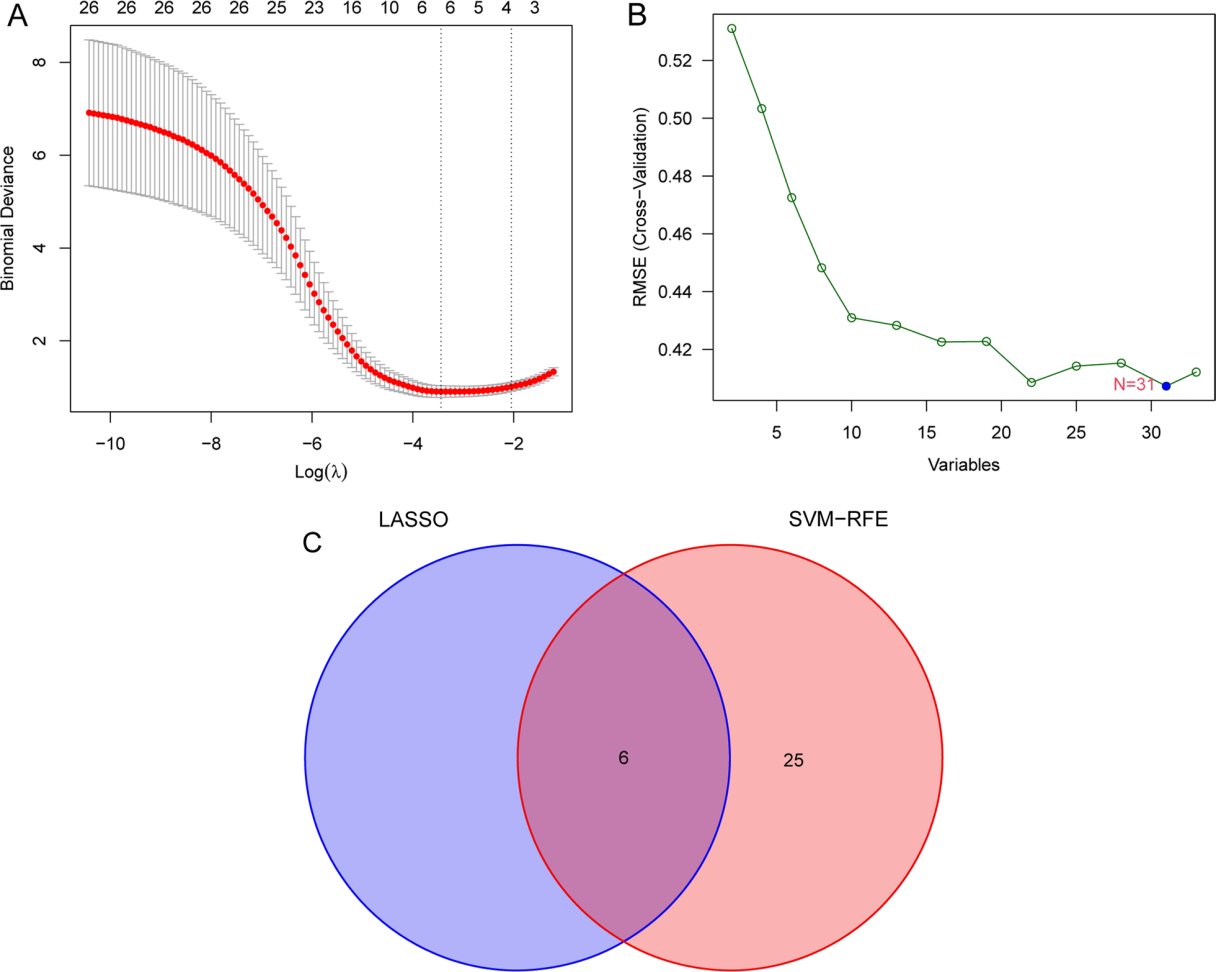
Procedure of screening potential diagnostic biomarkers for OS. **A** Tuning feature selection in the LASSO. **B** A plot of biomarkers selection via SVM–RFE algorithm. **C** The LASSO and SVM–RFE diagnostic processes have four diagnostic indicators in common, as shown in the Venn diagram below

**Fig. 3:**
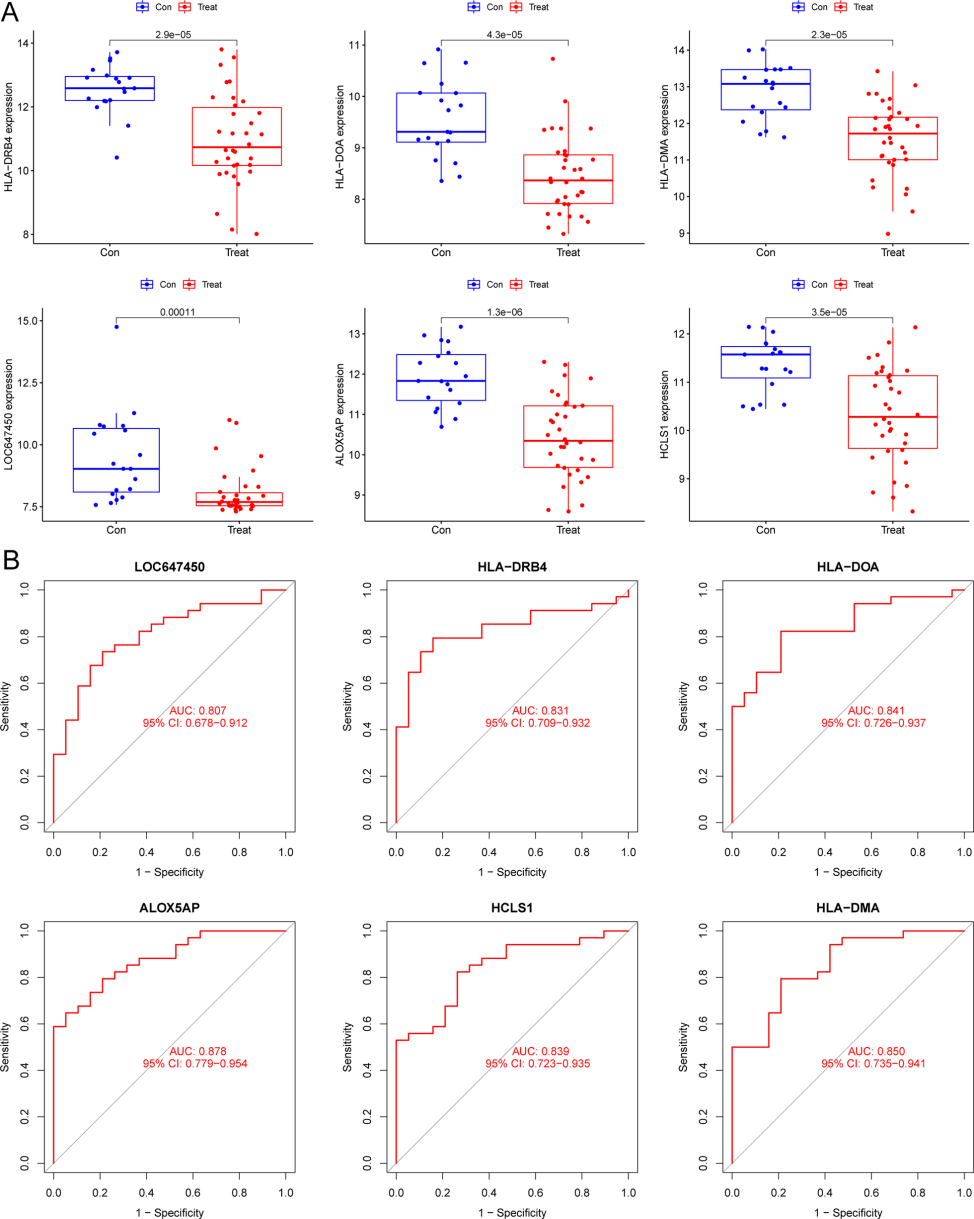
The expression pattern and diagnostic value of the critical gens for OS. **A**The expression of LOX5AP, HLA-DOA, HLA-DMA, HLA-DRB4, HCLS1 and LOC647450 between OS samples with metastasis and OS samples with non-metastasis. **B** The ROC assays of the diagnostic effectiveness of ALOX5AP, HLA-DOA, HLA-DMA, HLA-DRB4, HCLS1 and LOC647450

Two distinct methods were used in the quest for useful biomarkers. The DEGs were whittled down using the LASSO regression technique, and six variables were shown to be diagnostic biomarkers for OS (Fig. 2A). Using the SVM–RFE technique, a selection of 31 features from the DEGs was identified as being relevant for analysis (Fig. 2B). The six overlapping features (ALOX5AP, HLA-DOA, HLA-DMA, HLA-DRB4, HCLS1 and LOC647450) were finally identified (Fig. 2C). The expressing pattern of six genes is shown in Fig. 3A. We can observe that their expressions were distinctly decreased in OS specimens with metastasis. In addition, we performed ROC assays to examine the diagnostic value of the six genes and found that they showed a strong ability in screening OS specimens with metastasis from OS specimens with non-metastasis, with AUC > 0.7 (Fig. 3B).

### Relationship between the expression of six diagnostic genes and immunity

**Fig. 4:**
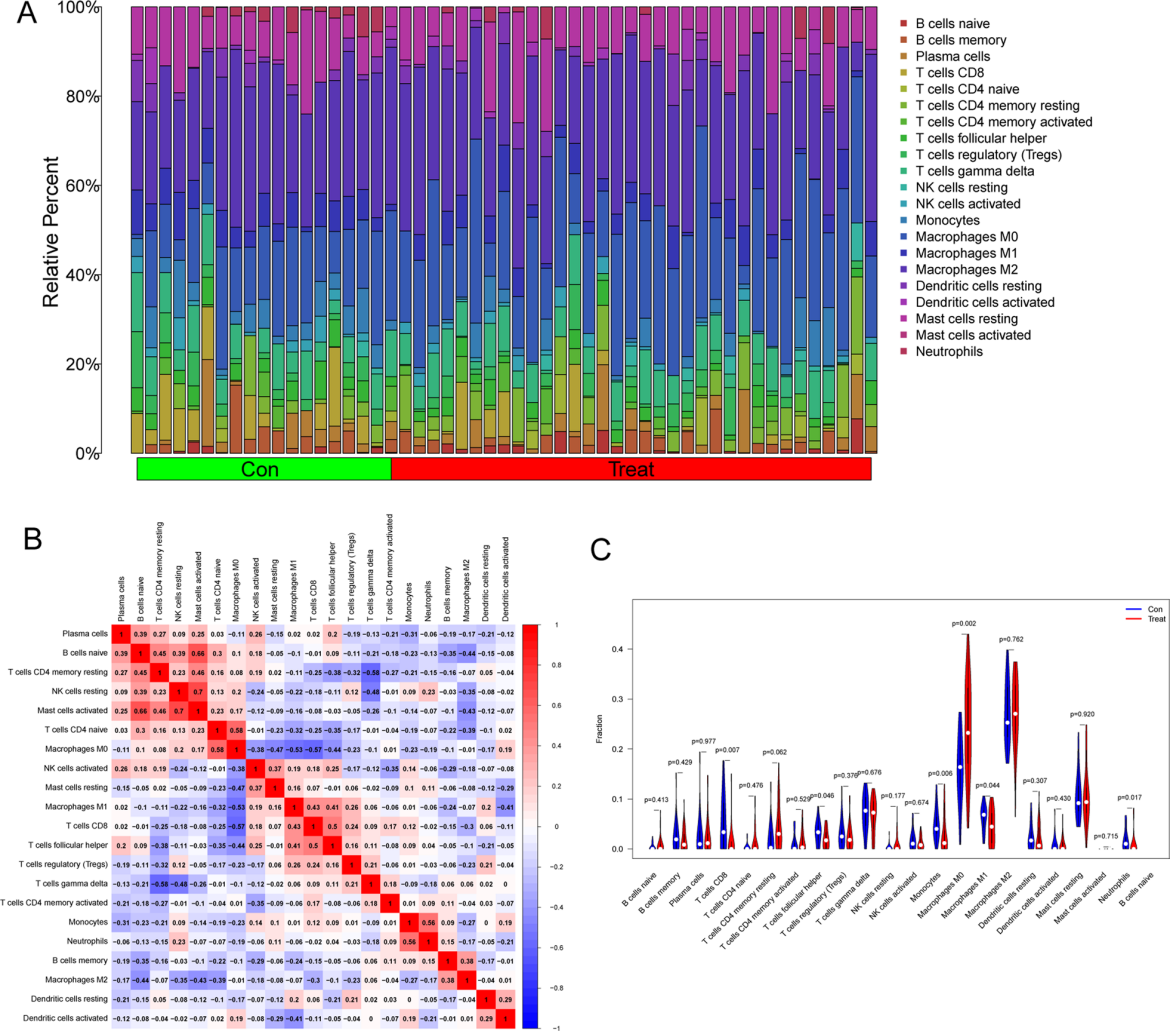
Analysis on the expressions of 22 TIICs and their associations in OS samples from GSE21257. **A** Through the examination of the TCGA database, the Heatmap of 22 TIICs, and immune cells among OS cases. **B** The matrix consisting of 22 different types of TIICs in OS was analyzed using Pearson's correlation coefficient. **C** The dissimilarity in the composition of TIICs between OS samples with metastasis and OS samples with non-metastasis

**Fig. 5:**
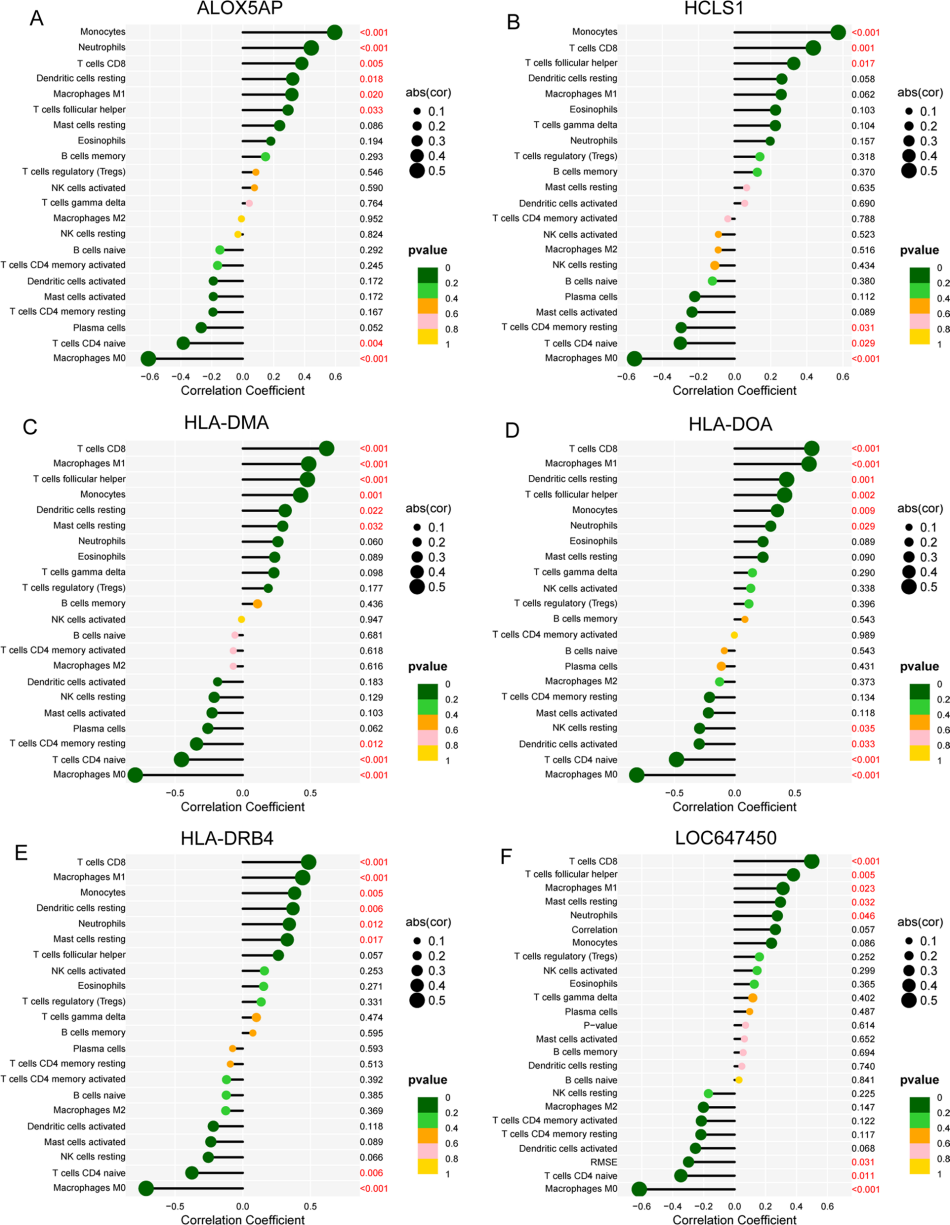
Correlation between **A** ALOX5AP, **B** HCLS1, **C** HLA-DMA, **D** HLA-DOA, **E** HLA-DRB4 and **F** LOC647450, and infiltrating immune cells in OS

Figure 4A shows the composition of tumor-infiltrating immune cells (TIICs) as well as the correlation between immune cells in OS specimens. We also proved the positive associations between each type of TIICs (Fig. 4B). In addition, we found significant differences in TIICs composition between OS specimens with metastasis and OS specimens with on-metastasis (Fig. 4C). Importantly, we observed that ALOX5AP was positively associated with monocytes, neutrophils, T cells CD8, dendritic cells resting, macrophages M1 and T cells follicular helper, while negatively associated with T cells CD4 naïve and Macrophages M0 (Fig. 5A). Moreover, the association between the other five genes, including HLA-DOA, HLA-DMA, HLA-DRB4, HCLS1 and LOC647450, is shown in Fig. 5B–F. Our findings indicate that the six diagnostic genes may play a role in modulating immune activity by regulating distinct signaling pathways, potentially contributing to disease progression.

### ALOX5AP expression in our cohort and cell lines

**Fig. 6:**
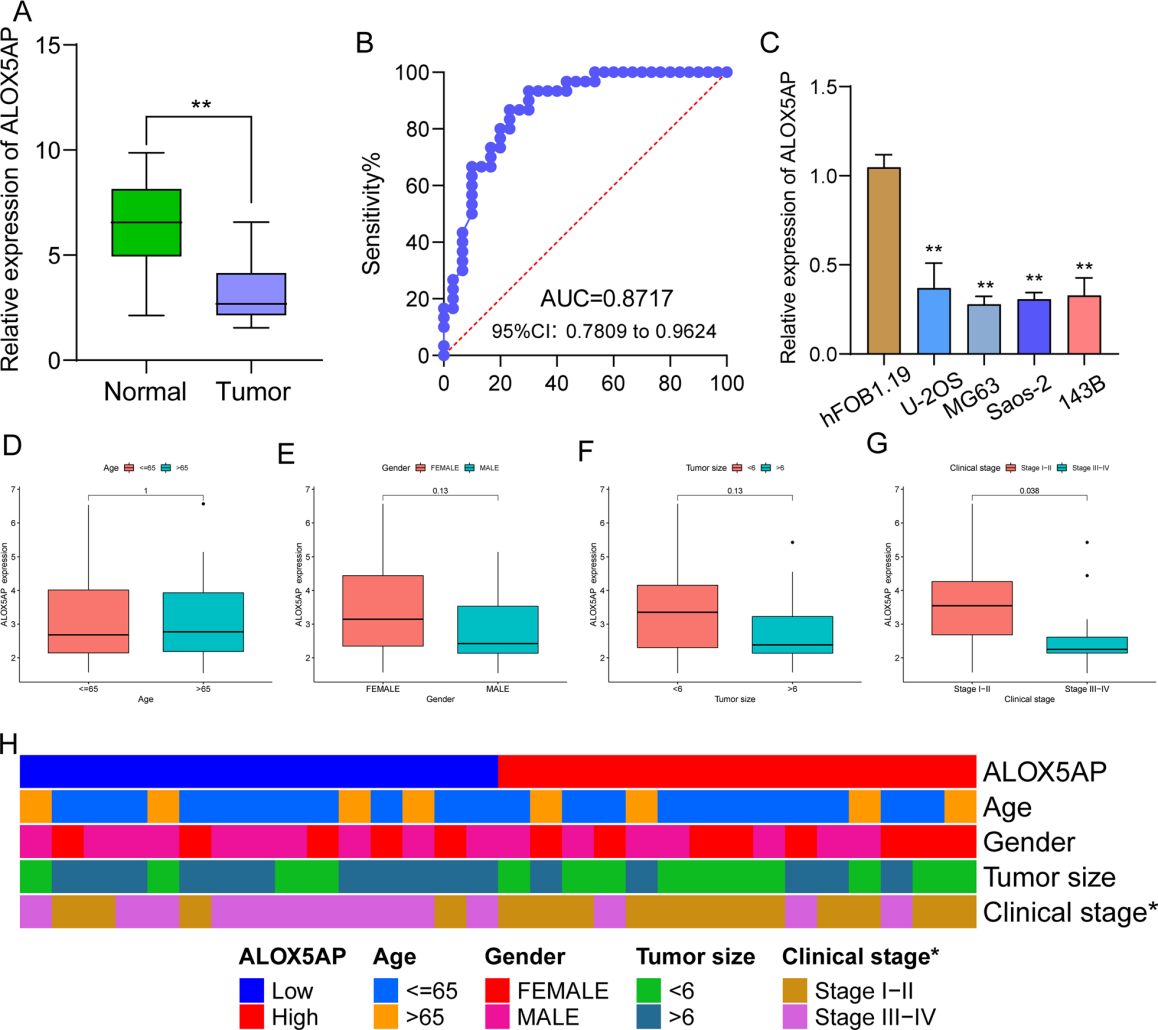
ALOX5AP was lowly expressed in OS and associated with advanced clinical stage. **A** RT-PCR was applied to examine the expression of ALOX5AP in OS specimens and non-tumor specimens. **B** ROC assays were applied to explore the diagnostic value of ALOX5AP expression in screening OS specimens from non-tumor specimens. **C** RT-PCR for the expression of ALOX5AP in four OS cell lines and hFOB1.19 cells. **D**–**G** Association between the expression of ALOX5AP and clinical characteristics in OS patients. **H** The association between ALOX5AP expression and different clinical factors are shown using heat map

Since there were limited reports on the expression and function of ALOX5AP in OS, we directed our attention toward investigating this gene. Then, we performed RT-PCR to examine the expression of ALOX5AP in 30 pairs of OS specimens and matched non-tumor specimens. As shown in Fig. 6A, the expression of ALOX5AP was distinctly downregulated in OS specimens compared with non-tumor specimens. In addition, the results of ROC assays confirmed the diagnostic value of ALOX5AP in screening OS specimens from non-tumor specimens with AUC = 0.8717 (Fig. 6B). Moreover, we also confirmed that ALOX5AP expression was distinctly increased in four OS cell lines compared with hFOB 1.19 (Fig. 6C). Then, we analyzed whether ALOX5AP exhibited a dysregulated level in OS specimens with different clinical characteristic. We found that ALOX5AP expression was not associated with age, gender and tumor size (Fig. 6D–F). However, we found that high ALOX5AP expression predicated an advanced clinical stage (Fig. 6G). The association between ALOX5AP expression and clinical factors is shown in heat Map (Fig. 6H).

### The prognostic value of ALOX5AP expression in OS patients

**Fig. 7:**
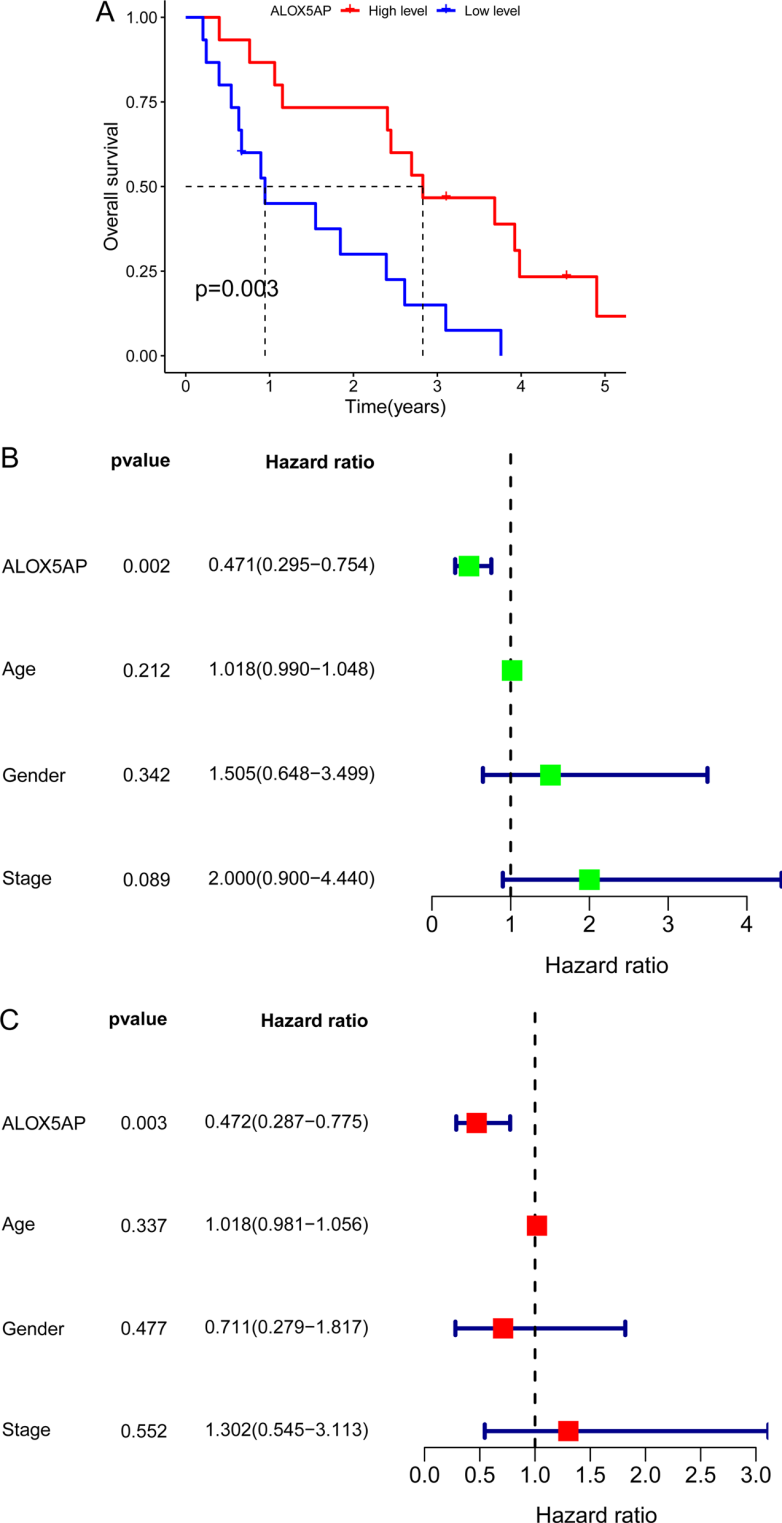
The prognostic value of ALOX5AP expression in OS patients. **A** Evaluation of the correlation between ALOX5AP expression and the overall survival of patients with OS. **B** Univariate and **C** multivariate analyses for overall survival in OS patients

By categorizing OS patients into high and low groups based on the mean expression of ALOX5AP, we conducted an investigation to determine whether this gene was associated with specific clinicopathological characteristics of OS. Overall survival was considerably poorer in patients with low ALOX5AP levels compared to those with high ALOX5AP levels, as seen by the Kaplan–Meier survival curves (*p* = 0.003; Fig. 7A). ALOX5AP expression was found to have a significant impact on overall survival in a univariate Cox regression analysis (Fig. 7B). Also, multivariate COX regression analysis confirmed that ALOX5AP expression levels were independent prognostic factors for overall survival (HR = 0.472, 95% CI 0.287–0.775, *p* = 0.003, Fig. 7C).

### *Knockdown of ALOX5AP suppresses OS cell proliferation *in vitro* and *in vivo

**Fig. 8:**
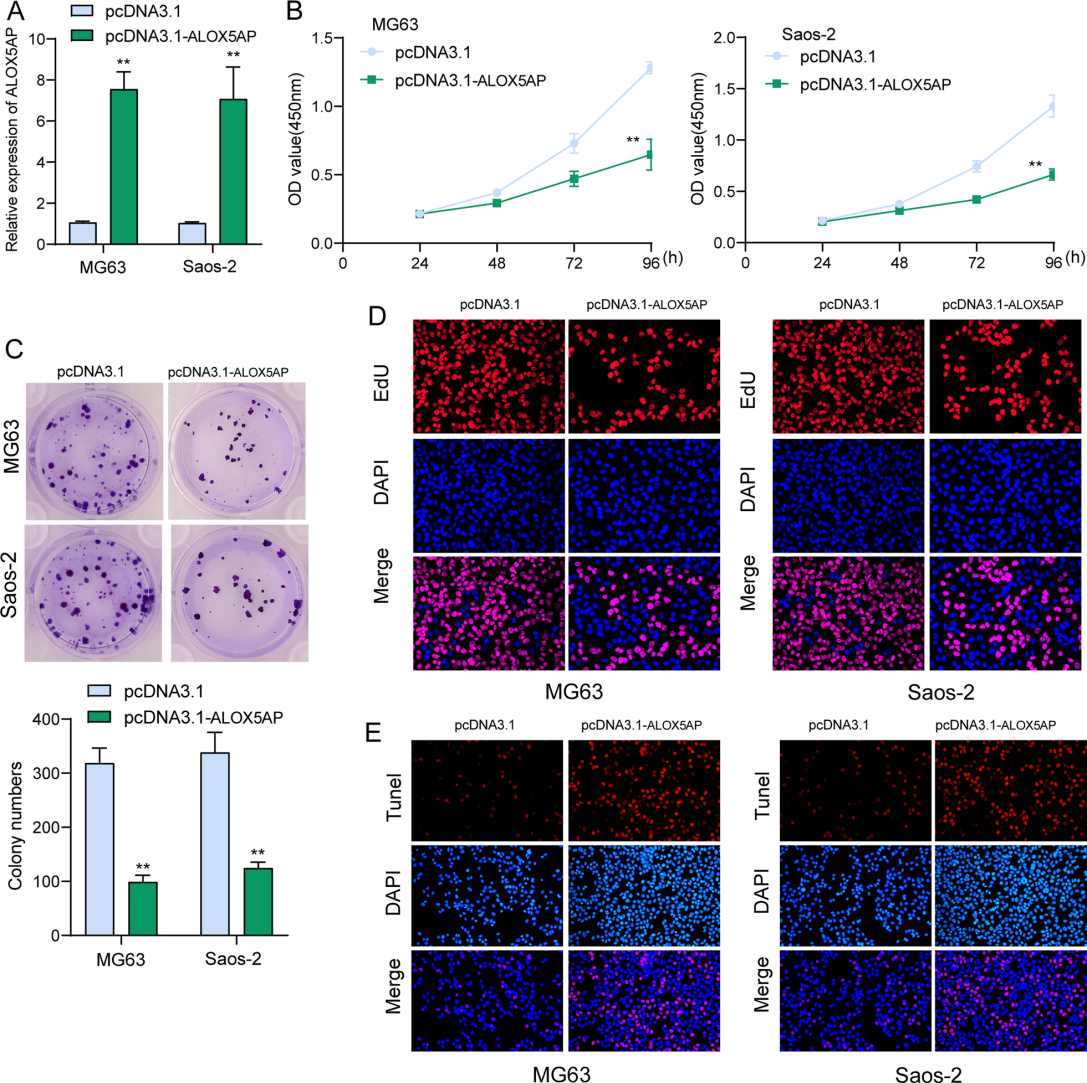
LOX5AP overexpression suppressed the proliferation of OS cells. **A** RT-PCR verified the sustained overexpression of ALOX5AP in MG63 and Saos-2 cells. **B**–**D** The proliferation rate status of MG63 and Saos-2 cells following ALOX5AP overexpression was determined using CCK-8, colony formation and EdU assays. **E** The ALOX5AP overexpression-induced apoptosis rate in MG63 and Saos-2 cells was analyzed using the Tunel assay

**Fig. 9:**
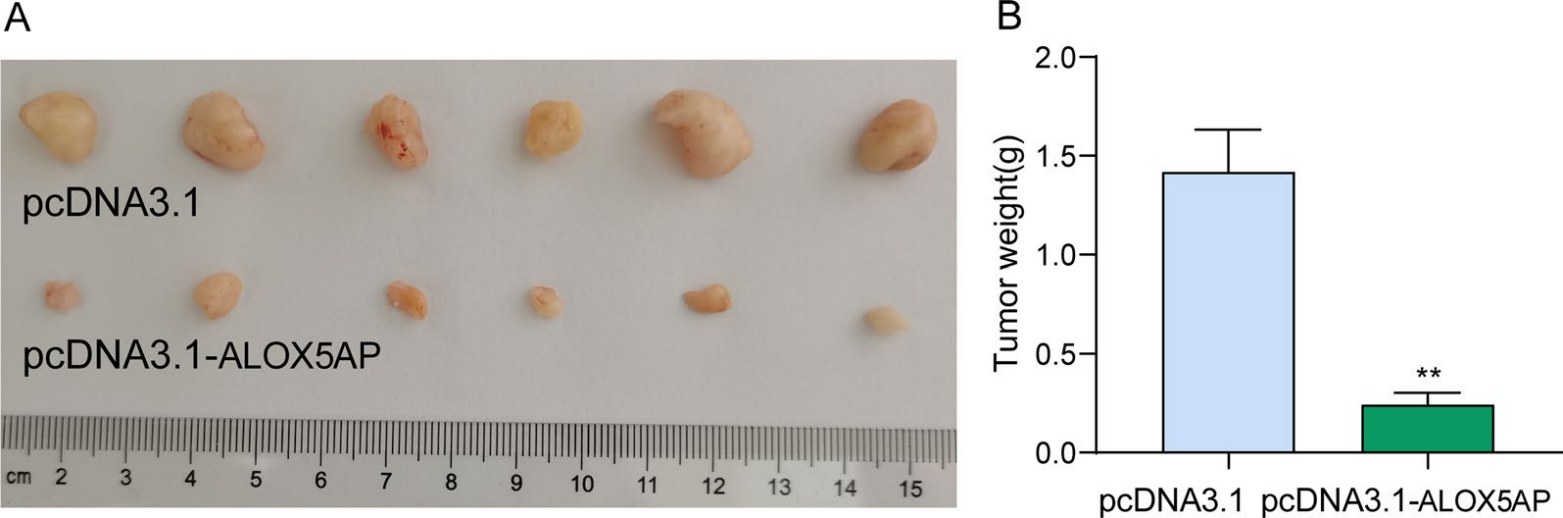
ALOX5AP overexpression inhibits tumor growth in vivo. **A** Representative images of xenograft tumors derived from ALOX5AP-overexpressing and vector control MG63 cells that were subcutaneously injected into Balb/c athymic nude mice. **B** Tumor weights were measured after the tumors were harvested from indicated groups

In order to investigate the possible biological function of ALOX5AP in OS cells, the expression levels of ALOX5AP were measured in two OS cell lines, MG63 and Saos-2. These cell lines had a lower level of ALOX5AP expression, and thus they were used to study the effects of pcDNA3.1-ALOX5AP-mediated overexpression of ALOX5AP on cell proliferation. PcDNA3.1-ALOX5AP constructs were employed to examine the level of overexpression, and the results demonstrated a significant increase in the expression of ALOX5AP upon transfection with pcDNA3.1-ALOX5AP in both MG63 and Saos-2 cells (Fig. 8A). According to the findings of CCK-8 tests, EdU staining and clonogenic assays, ALOX5AP overexpression significantly inhibited the proliferation of MG63 and Saos-2 cells (Fig. 8B–D). In addition, TUNEL experiments demonstrated that an increase in ALOX5AP levels greatly enhanced OS cell apoptosis (Fig. 8E). In addition to that, we also carried out in vivo assays. When compared to the pcDNA3.1-ALOX5AP groups, the tumors observed in the pcDNA3.1 groups were noticeably more extensive (Fig. 9A). Furthermore, ALOX5AP overexpression led to dramatically reduced tumor weights (Fig. 9B). These results suggested that ALOX5AP overexpression slowed the development of OS tumors in animal models.

### *ALOX5AP inhibited the migration and invasion of OS cells *via* WNT/β‐catenin signaling*

**Fig. 10:**
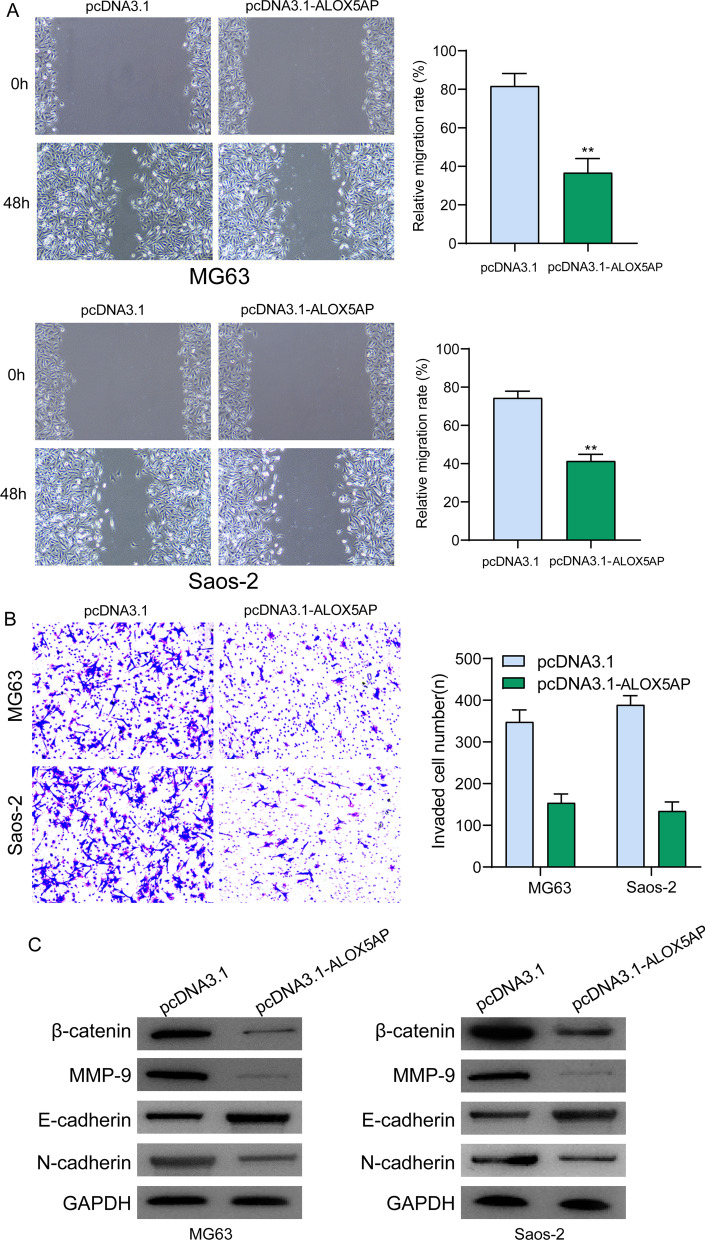
ALOX5AP overexpression suppressed cell migration and invasion of OS cells via regulating EMT and Wnt/β-catenin pathway. **A** Images taken with a representative microscope demonstrating the filling of wound lines by a wound-healing experiment in transfected MG63 and Saos-2 cells at 0 and 48 h after the transfection. **B** In order to examine the proliferation and migration of MG63 and Saos-2 cells, transwell migration assays were utilized. **C** After ALOX5AP overexpression, western blot analysis was used to examine the levels of N-cadherin, E-cadherin, MMP9 and Wnt/β-catenin pathway in MG63 and Saos-2 cells

We tested the effect of pcDNA3.1-ALOX5AP on the motility of MG63 and Saos-2 cells in order to learn more about the biological roles of ALOX5AP in OS. Wound-healing experiments showed that after ALOX5AP overexpression, OS cell migration was significantly reduced (Fig. 10A). Transwell assays were also used to investigate the effects on cellular invasiveness. The results indicated that ALOX5AP overexpression significantly reduced OS cells' invasive potential (Fig. 10B). To further investigate the impact of ALOX5AP overexpression on the EMT pathway, we used western blotting. As shown in Fig. 10C, overexpressing ALOX5AP in MG63 and Saos-2 cells led to a substantial upregulation of E-cadherin expression and a downregulation of N-cadherin expression. In addition, we investigated the activity of the Wnt/β‐catenin signaling pathway in order to identify the underlying molecular processes that are responsible for the suppressive effects of ALOX5AP overexpression on OS cell proliferation, migration and invasion. Western blotting revealed that ALOX5AP overexpression in MG63 and Saos-2 cells significantly suppressed the production of β-catenin and MMP9, two effectors of the Wnt/β‐catenin pathway (Fig. 10C).

## Discussion

The mortality rate of cancer patients is gradually reducing as a result of continual advances in surgical procedures and the continuous introduction of various therapeutic treatments [17, 18]. On the other hand, the overall survival rate of OS patients has reached a bottleneck phase since the 1970s, and it has not improved to this day [19]. Developing personalized treatment regimens through graded management is of utmost importance for cancer patients, as it is expected to significantly improve their prognosis [20, 21]. The continual growth of the idea of precision medicine has made this particularly crucial. The evidence that genetic alterations and epigenetic modifications play a crucial role in the developments of cancers is growing stronger all the time [22, 23]. In clinical settings, the utilization of genetic testing to assess patients' prognoses, particularly in relation to their response to medication treatment, has recently commenced. However, the majority of these tests are quite pricey and require tissue from the patients.

In recent years, the development of machine learning provided novel methods for the identification of novel biomarkers for various diseases. In this study, we firstly identified 33 DEGs: 33 genes were significantly downregulated. Then, we used LASSO and SVM algorithms to screen critical diagnostic genes for metastasis OS. Importantly, we identified six critical diagnostic gene, including ALOX5AP, HLA-DOA, HLA-DMA, HLA-DRB4, HCLS1 and LOC647450. The function of the above genes has been reported in several tumors. For instance, Ye et al. reported that when compared to levels in normal tissues, the expressions of ALOX5AP mRNA found in serous ovarian cancer tissues were considerably elevated. In different serous ovarian cancer patient cohorts, an elevated level of ALOX5AP was significantly related not only to a lower overall survival and progression-free survival, but also to unfavorable clinicopathological characteristics. ALOX5AP exhibited significant expression in the immunoreactive subtype of ovarian cancer, and its expression was directly correlated with immunocyte infiltration, particularly the polarization of M2 macrophages [24]. Chen et al. reported that ALOX5AP was associated with clinical prognosis of OS patients and was identified as an indicator of TME in OS based on bioinformatics analysis. They did not perform clinical experiments to confirm their findings [25]. In addition, the potential of ALOX5AP used as a novel biomarker was also demonstrated in a previous study [26]. However, the expression and function of ALOX5AP, HLA-DOA, HLA-DMA, HLA-DRB4, HCLS1 and LOC647450 in OS was rarely reported. In this research, we confirmed their expressions were distinctly decreased in OS specimens with metastasis compared with OS specimens with non-metastasis, suggesting that they may be involved in the progression of distant metastasis.

The immunological microenvironment is composed of a wide variety of immune cells as well as chemicals that are secreted [27]. It is possible that immunological capabilities, the invasion of tumor cells and the activation of targeted therapies are all factors that can decide the outcomes for cancer patients and foretell how well they will respond to immunotherapies [28, 29]. Certain functional genes possess the potential to induce modifications in the immunological milieu, as well as influence immune-related metabolic pathways [30, 31]. In this study, we observed that ALOX5AP was positively associated with monocytes, neutrophils, T cells CD8, dendritic cells resting, macrophages M1 and T cells follicular helper, while negatively associated with T cells CD4 naïve and Macrophages M0. In addition, the other five genes were also observed to be associated with several immune cells, especially T cells CD8. Many studies have confirmed the positive roles of T cells CD8 in tumor progression. Our findings suggested ALOX5AP, HLA-DOA, HLA-DMA, HLA-DRB4, HCLS1 and LOC647450 may be protected genes for metastasis via regulating T cells CD8.

In the end, we settled on ALOX5AP for more research. Expression of ALOX5AP was shown to be significantly lower in OS samples compared to non-tumor controls by RT-PCR. Clinical tests indicated that lower levels of ALOX5AP expression were correlated with a more severe clinical presentation and an unfavorable prognosis. The potential of ALOX5AP as a new biomarker for OS patients was underscored by the fact that multivariate analysis indicated that it was an independent predictive factor for overall survival of OS patients. In terms of function, we discovered that ALOX5AP overexpression markedly inhibited OS cell proliferation, migration, invasion and the EMT process. The Wnt/β‐catenin signaling system is a conserved signaling axis involved in a wide variety of physiological activities, including cell proliferation, differentiation, apoptosis, migration, invasion and tissue homeostasis [32–34]. Several types of cancer, including solid tumors and hematological malignancies, have been linked to Wnt/β‐catenin cascade dysregulation [35–37]. In this study, we also examined whether ALOX5AP exhibited its tumor-suppressive roles via regulating Wnt/β-catenin pathway. Interestingly, we observed that the expression of β-catenin and MMP9, which are the effectors of Wnt/β-catenin pathway, was obviously downregulated after ALOX5AP overexpression in MG63 and Saos-2 cells. Thus, our finding suggested ALOX5AP suppressed OS progression via regulating Wnt/β-catenin pathway.

## Conclusion

Overall, using bioinformatics techniques and two machine-learning algorithms, we identified 6 characteristic genes of OS. We also investigated the biological functions and pathways that were associated with these genes. Notably, the specific genes that were tested and validated in our research may be associated with varying levels of immune infiltration in patients with OS. Among six critical genes, we further confirmed that ALOX5AP was lowly expressed in OS and predicted a favorable prognosis. In functional assays, we confirmed that ALOX5AP served as a tumor suppressor in OS via regulating Wnt/β-catenin pathway. To make substantial progress in the battle against cancer, it is essential to enhance our understanding of the immunological status of the disease. This approach may provide some reference values that can be used to generate solid criteria for medication selection, boost treatment responses and help doctors manage patients with OS.

## Data Availability

According to the requirements, data and materials can be obtained from the corresponding authors to support the results of this study.
